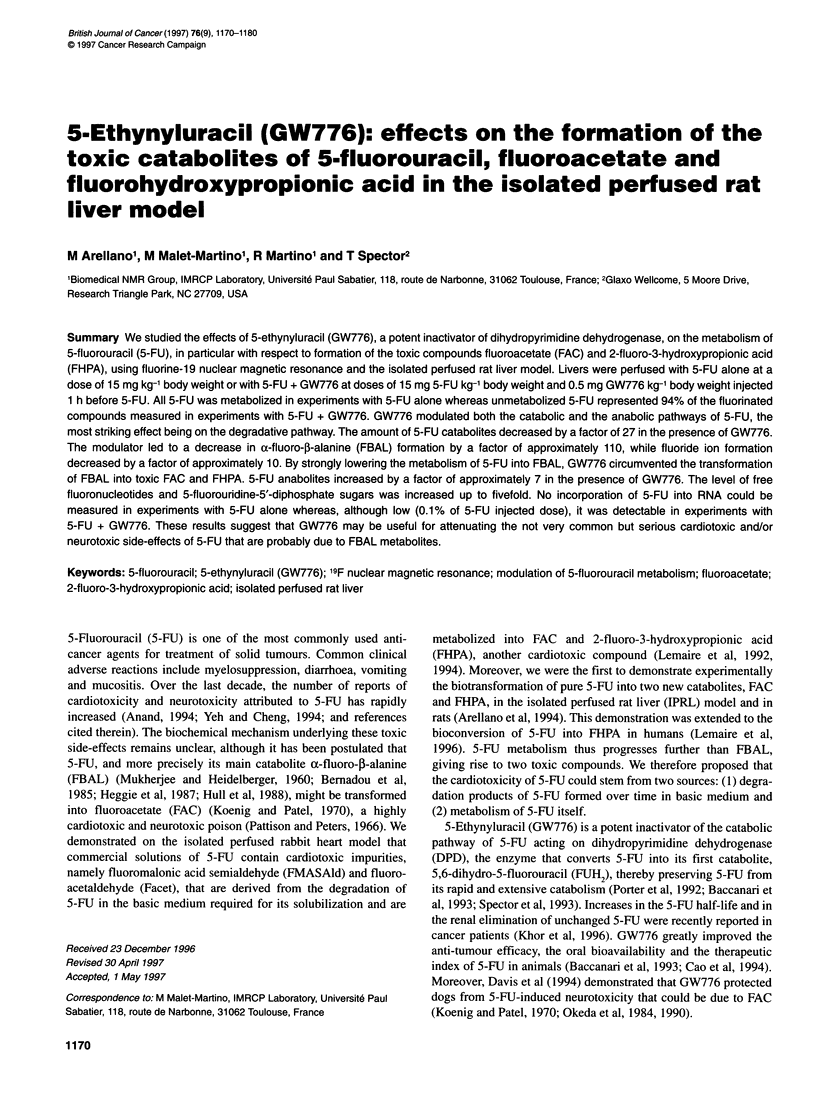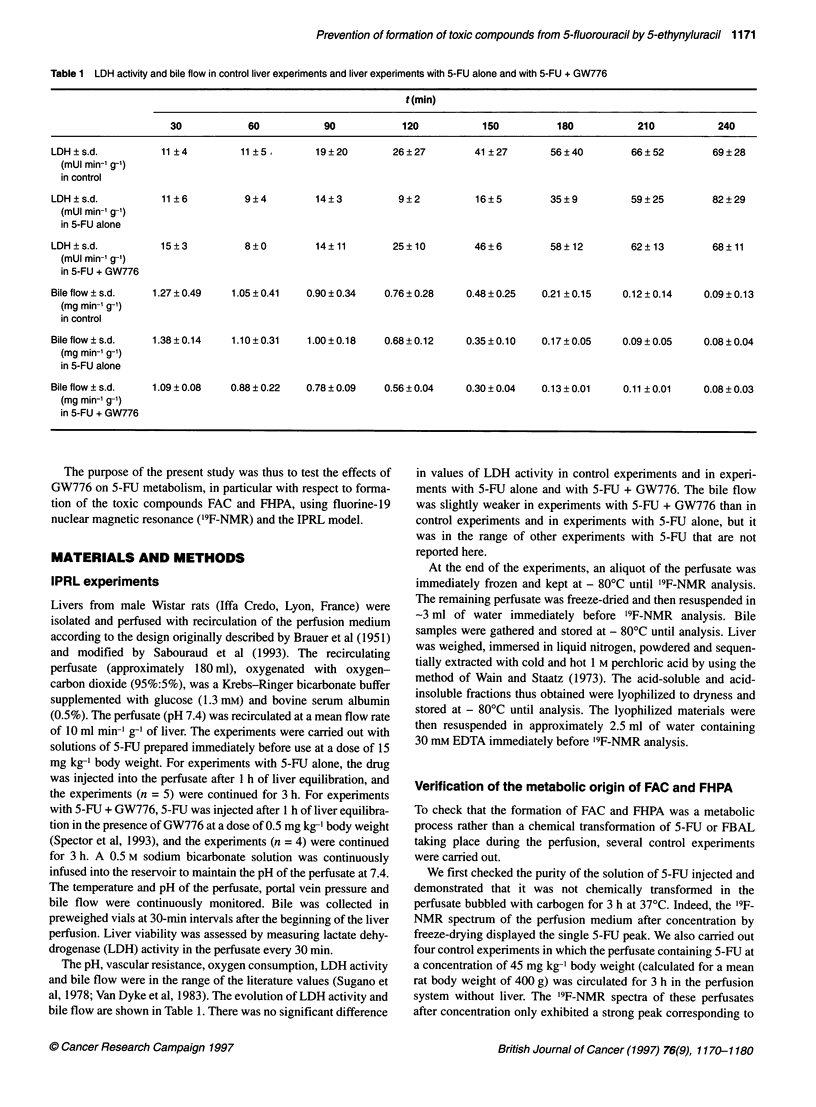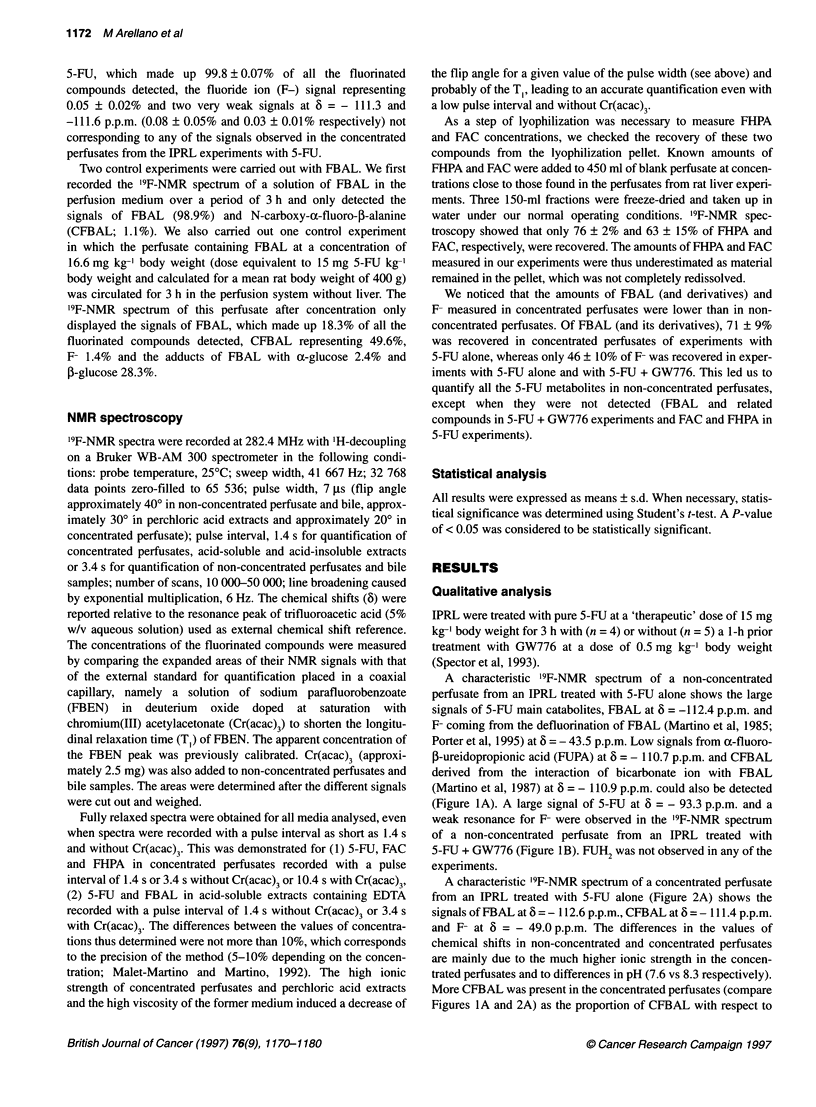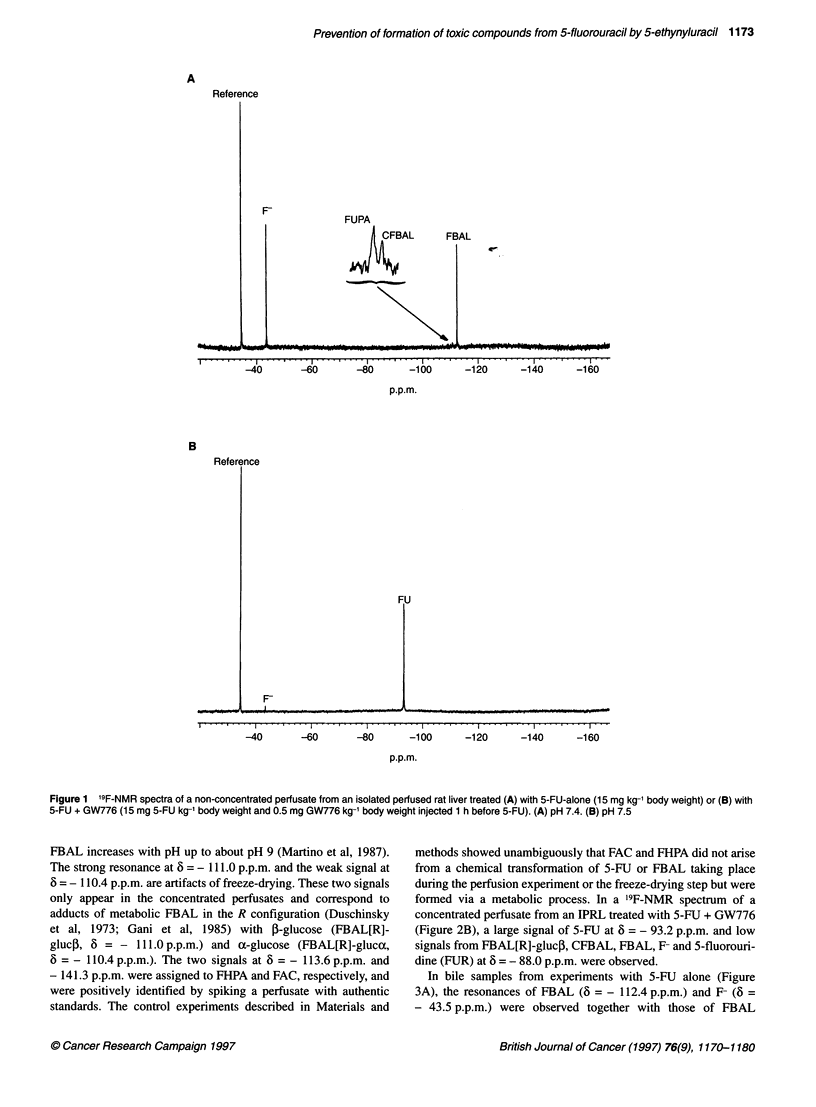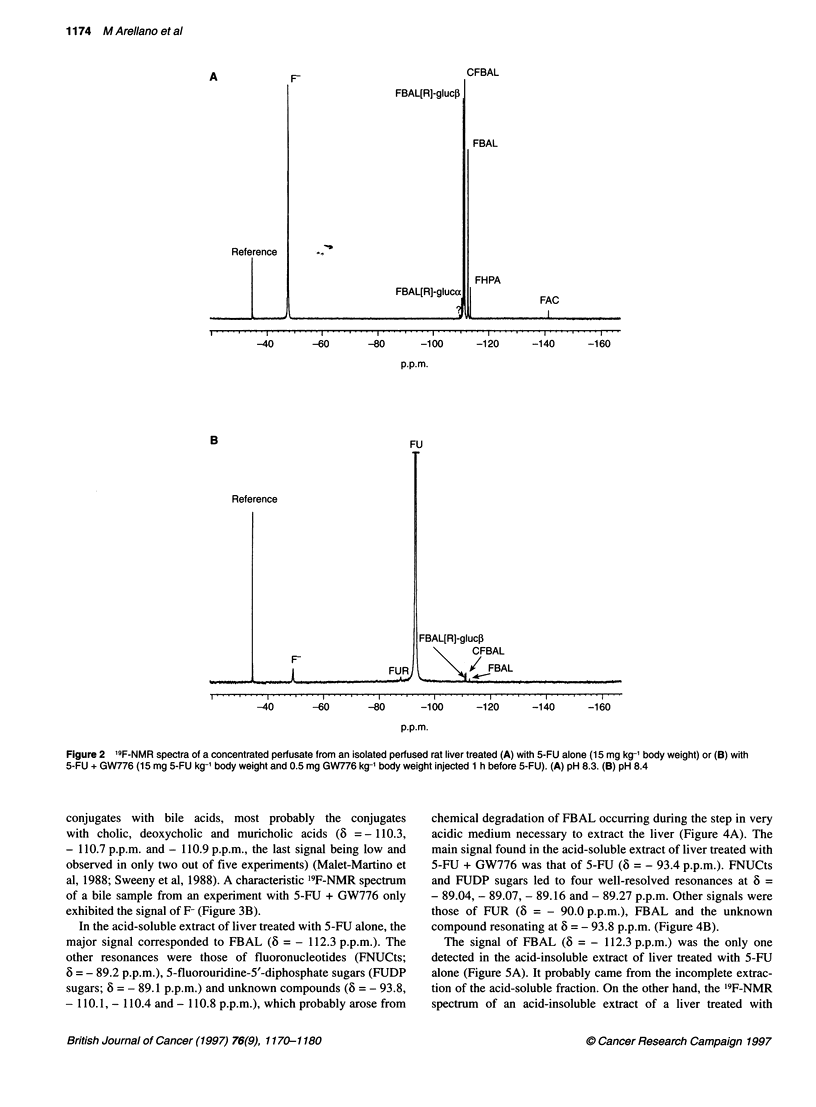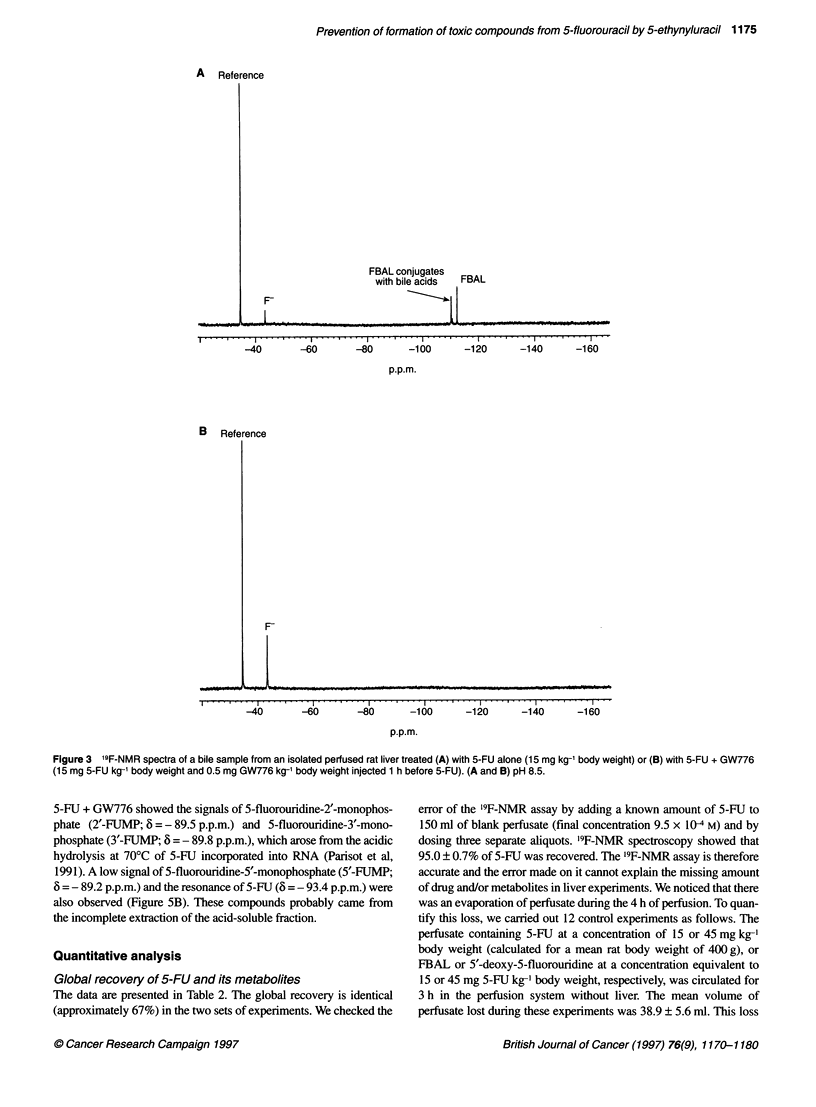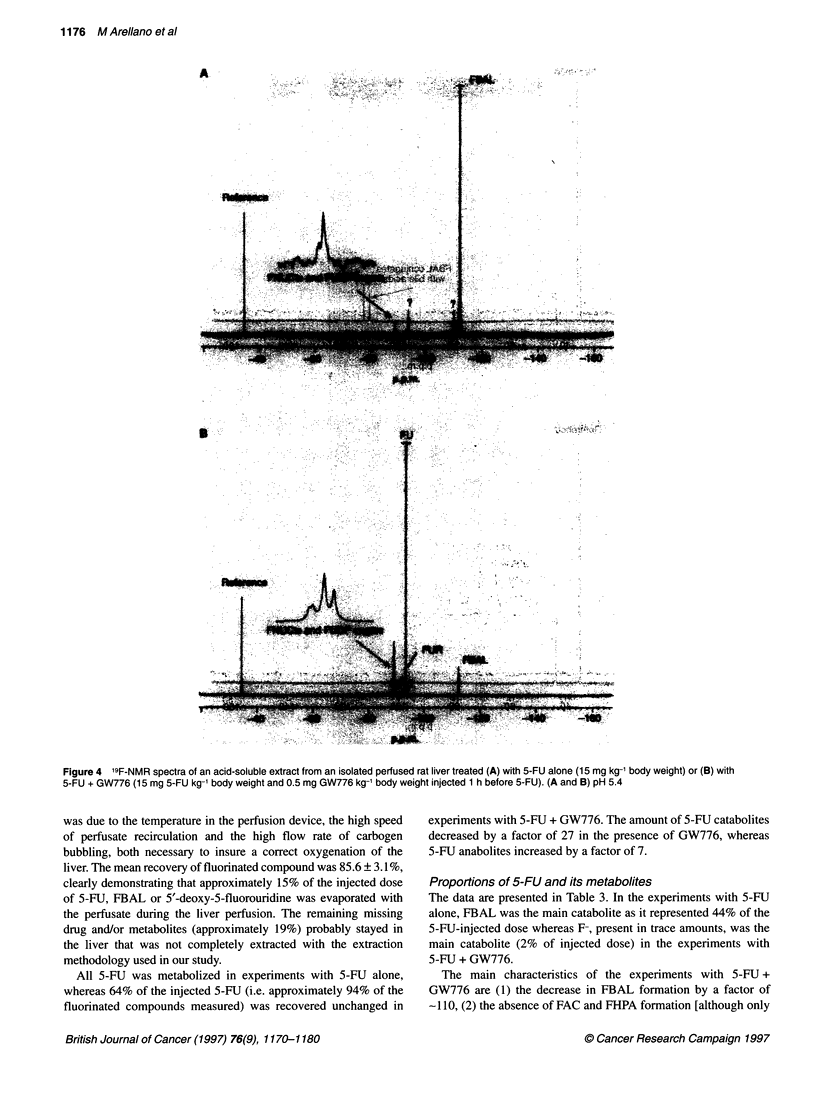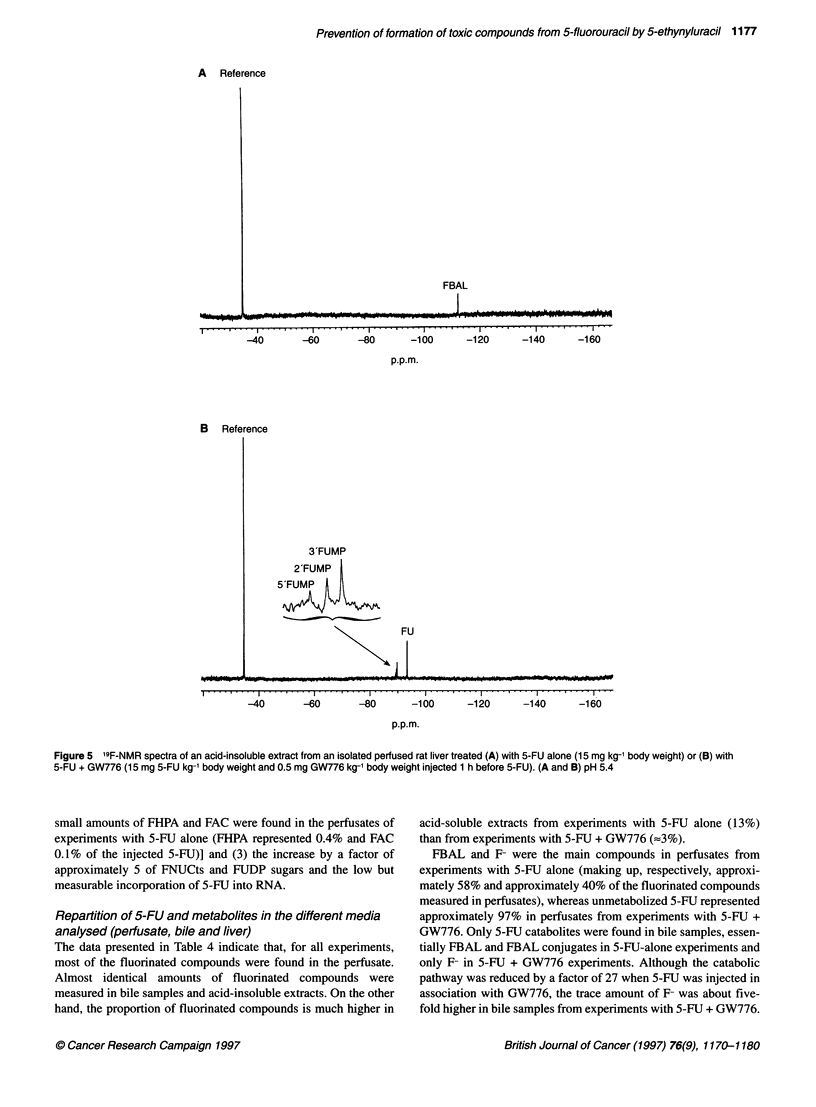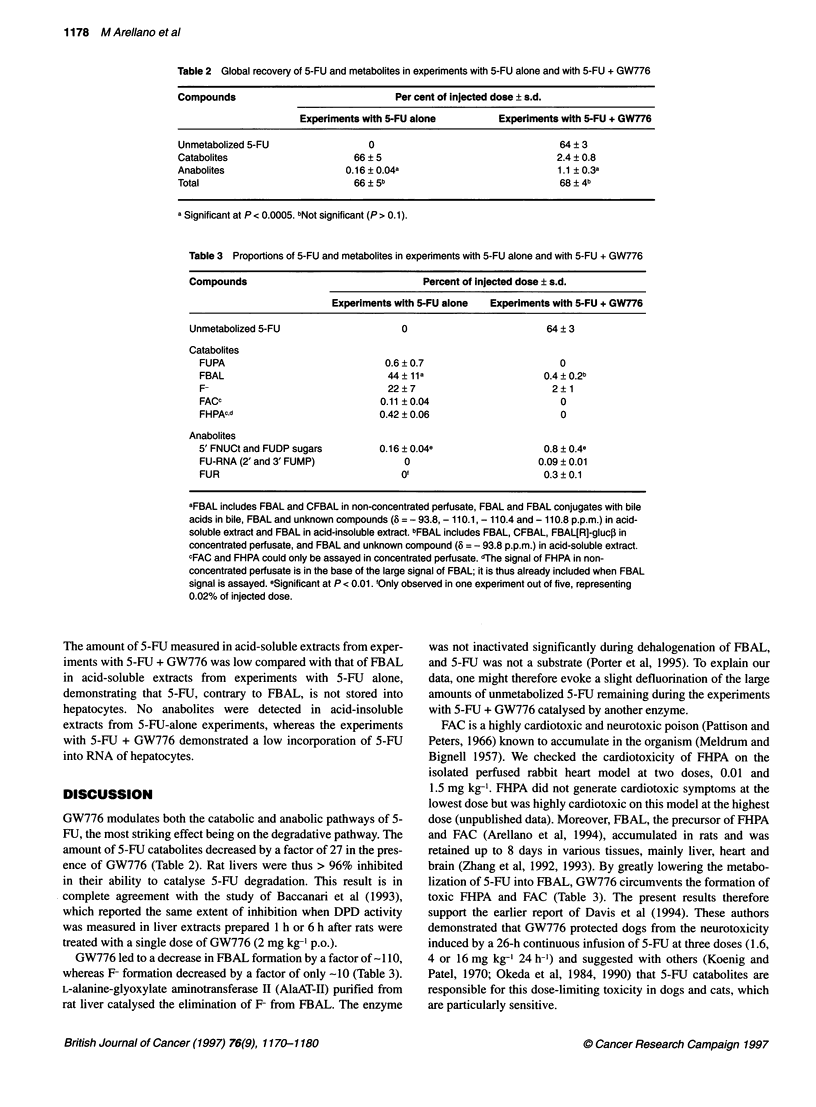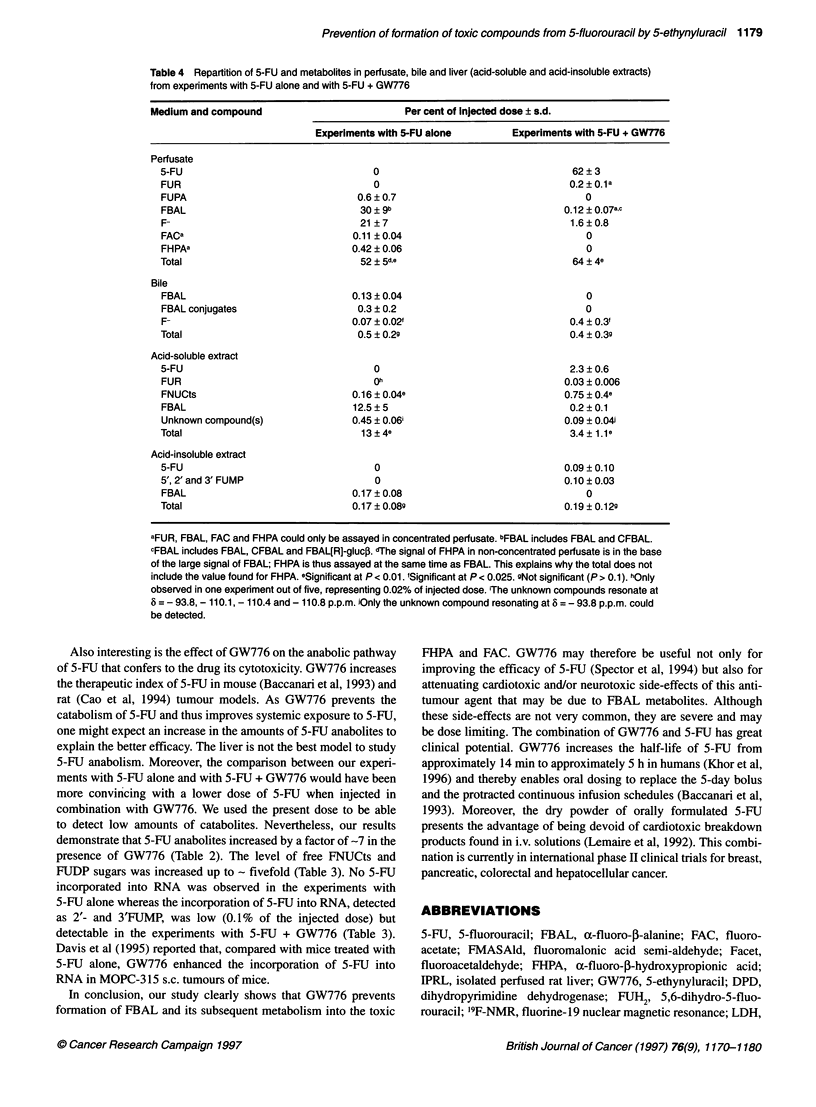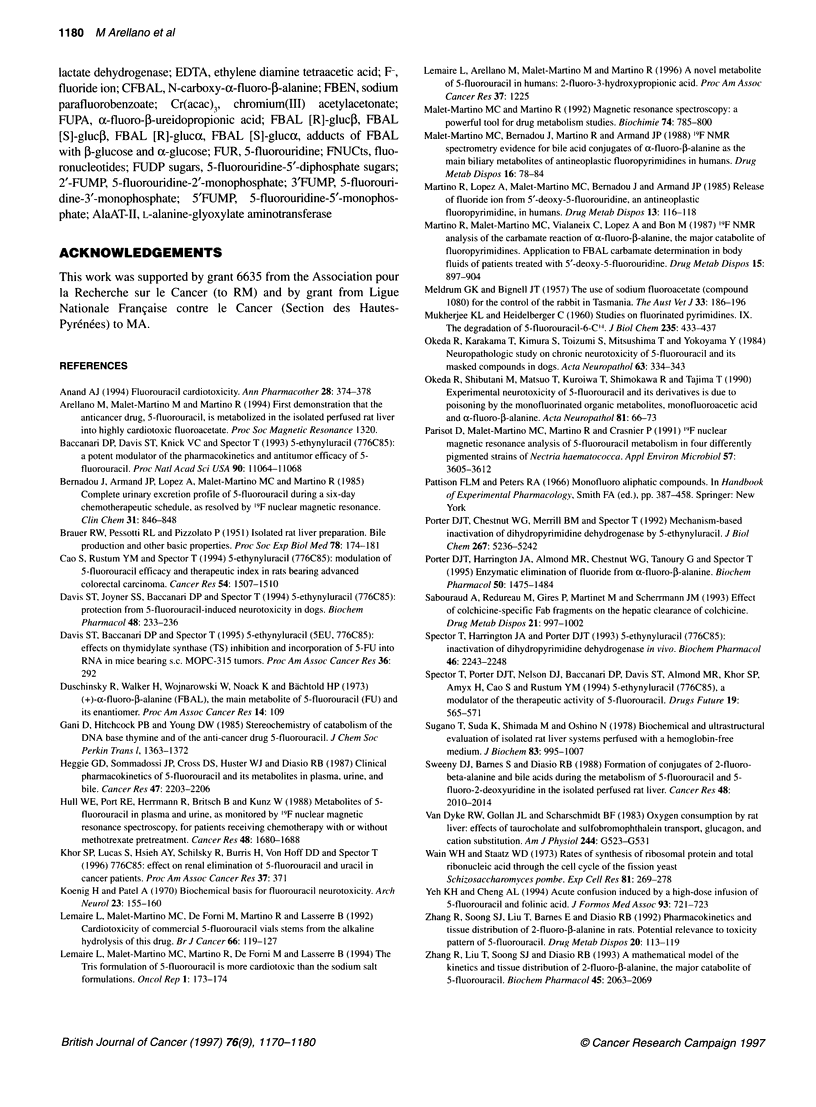# 5-Ethynyluracil (GW776): effects on the formation of the toxic catabolites of 5-fluorouracil, fluoroacetate and fluorohydroxypropionic acid in the isolated perfused rat liver model.

**DOI:** 10.1038/bjc.1997.529

**Published:** 1997

**Authors:** M. Arellano, M. Malet-Martino, R. Martino, T. Spector

**Affiliations:** Biomedical NMR Group, IMRCP Laboratory, UniversitÃ© Paul Sabatier, Toulouse, France.

## Abstract

We studied the effects of 5-ethynyluracil (GW776), a potent inactivator of dihydropyrimidine dehydrogenase, on the metabolism of 5-fluorouracil (5-FU), in particular with respect to formation of the toxic compounds fluoroacetate (FAC) and 2-fluoro-3-hydroxypropionic acid (FHPA), using fluorine-19 nuclear magnetic resonance and the isolated perfused rat liver model. Livers were perfused with 5-FU alone at a dose of 15 mg kg(-1) body weight or with 5-FU + GW776 at doses of 15 mg 5-FU kg(-1) body weight and 0.5 mg GW776 kg(-1) body weight injected 1 h before 5-FU. All 5-FU was metabolized in experiments with 5-FU alone whereas unmetabolized 5-FU represented 94% of the fluorinated compounds measured in experiments with 5-FU + GW776. GW776 modulated both the catabolic and the anabolic pathways of 5-FU, the most striking effect being on the degradative pathway. The amount of 5-FU catabolites decreased by a factor of 27 in the presence of GW776. The modulator led to a decrease in alpha-fluoro-beta-alanine (FBAL) formation by a factor of approximately 110, while fluoride ion formation decreased by a factor of approximately 10. By strongly lowering the metabolism of 5-FU into FBAL, GW776 circumvented the transformation of FBAL into toxic FAC and FHPA. 5-FU anabolites increased by a factor of approximately 7 in the presence of GW776. The level of free fluoronucleotides and 5-fluorouridine-5'-diphosphate sugars was increased up to fivefold. No incorporation of 5-FU into RNA could be measured in experiments with 5-FU alone whereas, although low (0.1% of 5-FU injected dose), it was detectable in experiments with 5-FU + GW776. These results suggest that GW776 may be useful for attenuating the not very common but serious cardiotoxic and/or neurotoxic side-effects of 5-FU that are probably due to FBAL metabolites.


British Journal of Cancer (1997) 76(9), 1170–1180
© 1997 Cancer Research Campaign

### 5-Ethynyluracil (GW776): effects on the formation of the toxic catabolites of 5-fluorouracil, fluoroacetate and fluorohydroxypropionic acid in the isolated perfused rat liver model

**M Arellano[1], M Malet-Martino[1], R Martino[1] and T Spector[2]**

[1]Biomedical NMR Group, IMRCP Laboratory, Université Paul Sabatier, 118, route de Narbonne, 31062 Toulouse, France; [2]Glaxo Wellcome, 5 Moore Drive, Research Triangle Park, NC 27709, USA

**Summary** We studied the effects of 5-ethynyluracil (GW776), a potent inactivator of dihydropyrimidine dehydrogenase, on the metabolism of 5-fluorouracil (5-FU), in particular with respect to formation of the toxic compounds fluoroacetate (FAC) and 2-fluoro-3-hydroxypropionic acid (FHPA), using fluorine-19 nuclear magnetic resonance and the isolated perfused rat liver model. Livers were perfused with 5-FU alone at a dose of 15 mg kg$^{-1}$ body weight or with 5-FU + GW776 at doses of 15 mg 5-FU kg$^{-1}$ body weight and 0.5 mg GW776 kg$^{-1}$ body weight injected 1 h before 5-FU. All 5-FU was metabolized in experiments with 5-FU alone whereas unmetabolized 5-FU represented 94% of the fluorinated compounds measured in experiments with 5-FU + GW776. GW776 modulated both the catabolic and the anabolic pathways of 5-FU, the most striking effect being on the degradative pathway. The amount of 5-FU catabolites decreased by a factor of 27 in the presence of GW776. The modulator led to a decrease in α-fluoro-β-alanine (FBAL) formation by a factor of approximately 110, while fluoride ion formation decreased by a factor of approximately 10. By strongly lowering the metabolism of 5-FU into FBAL, GW776 circumvented the transformation of FBAL into toxic FAC and FHPA. 5-FU anabolites increased by a factor of approximately 7 in the presence of GW776. The level of free fluoronucleotides and 5-fluorouridine-5′-diphosphate sugars was increased up to fivefold. No incorporation of 5-FU into RNA could be measured in experiments with 5-FU alone whereas, although low (0.1% of 5-FU injected dose), it was detectable in experiments with 5-FU + GW776. These results suggest that GW776 may be useful for attenuating the not very common but serious cardiotoxic and/or neurotoxic side-effects of 5-FU that are probably due to FBAL metabolites.

**Keywords:** 5-fluorouracil; 5-ethynyluracil (GW776); $^{19}$F nuclear magnetic resonance; modulation of 5-fluorouracil metabolism; fluoroacetate; 2-fluoro-3-hydroxypropionic acid; isolated perfused rat liver

5-Fluorouracil (5-FU) is one of the most commonly used anticancer agents for treatment of solid tumours. Common clinical adverse reactions include myelosuppression, diarrhoea, vomiting and mucositis. Over the last decade, the number of reports of cardiotoxicity and neurotoxicity attributed to 5-FU has rapidly increased (Anand, 1994; Yeh and Cheng, 1994; and references cited therein). The biochemical mechanism underlying these toxic side-effects remains unclear, although it has been postulated that 5-FU, and more precisely its main catabolite α-fluoro-β-alanine (FBAL) (Mukherjee and Heidelberger, 1960; Bernadou et al, 1985; Heggie et al, 1987; Hull et al, 1988), might be transformed into fluoroacetate (FAC) (Koenig and Patel, 1970), a highly cardiotoxic and neurotoxic poison (Pattison and Peters, 1966). We demonstrated on the isolated perfused rabbit heart model that commercial solutions of 5-FU contain cardiotoxic impurities, namely fluoromalonic acid semialdehyde (FMASAld) and fluoroacetaldehyde (Facet), that are derived from the degradation of 5-FU in the basic medium required for its solubilization and are metabolized into FAC and 2-fluoro-3-hydroxypropionic acid (FHPA), another cardiotoxic compound (Lemaire et al, 1992, 1994). Moreover, we were the first to demonstrate experimentally the biotransformation of pure 5-FU into two new catabolites, FAC and FHPA, in the isolated perfused rat liver (IPRL) model and in rats (Arellano et al, 1994). This demonstration was extended to the bioconversion of 5-FU into FHPA in humans (Lemaire et al, 1996). 5-FU metabolism thus progresses further than FBAL, giving rise to two toxic compounds. We therefore proposed that the cardiotoxicity of 5-FU could stem from two sources: (1) degradation products of 5-FU formed over time in basic medium and (2) metabolism of 5-FU itself.

5-Ethynyluracil (GW776) is a potent inactivator of the catabolic pathway of 5-FU acting on dihydropyrimidine dehydrogenase (DPD), the enzyme that converts 5-FU into its first catabolite, 5,6-dihydro-5-fluorouracil ($FUH_2$), thereby preserving 5-FU from its rapid and extensive catabolism (Porter et al, 1992; Baccanari et al, 1993; Spector et al, 1993). Increases in the 5-FU half-life and in the renal elimination of unchanged 5-FU were recently reported in cancer patients (Khor et al, 1996). GW776 greatly improved the anti-tumour efficacy, the oral bioavailability and the therapeutic index of 5-FU in animals (Baccanari et al, 1993; Cao et al, 1994). Moreover, Davis et al (1994) demonstrated that GW776 protected dogs from 5-FU-induced neurotoxicity that could be due to FAC (Koenig and Patel, 1970; Okeda et al, 1984, 1990).

*Received 23 December 1996*
*Revised 30 April 1997*
*Accepted, 1 May 1997*

*Correspondence to:* M Malet-Martino, IMRCP Laboratory, Université Paul Sabatier, 118, route de Narbonne, 31062 Toulouse, France

**Table 1** LDH activity and bile flow in control liver experiments and liver experiments with 5-FU alone and with 5-FU + GW776

| | *t* (min) | | | | | | | |
|---|---|---|---|---|---|---|---|---|
| | 30 | 60 | 90 | 120 | 150 | 180 | 210 | 240 |
| LDH ± s.d. (mUI $min^{-1}$ $g^{-1}$) in control | 11 ± 4 | 11 ± 5 | 19 ± 20 | 26 ± 27 | 41 ± 27 | 56 ± 40 | 66 ± 52 | 69 ± 28 |
| LDH ± s.d. (mUI $min^{-1}$ $g^{-1}$) in 5-FU alone | 11 ± 6 | 9 ± 4 | 14 ± 3 | 9 ± 2 | 16 ± 5 | 35 ± 9 | 59 ± 25 | 82 ± 29 |
| LDH ± s.d. (mUI $min^{-1}$ $g^{-1}$) in 5-FU + GW776 | 15 ± 3 | 8 ± 0 | 14 ± 11 | 25 ± 10 | 46 ± 6 | 58 ± 12 | 62 ± 13 | 68 ± 11 |
| Bile flow ± s.d. (mg $min^{-1}$ $g^{-1}$) in control | 1.27 ± 0.49 | 1.05 ± 0.41 | 0.90 ± 0.34 | 0.76 ± 0.28 | 0.48 ± 0.25 | 0.21 ± 0.15 | 0.12 ± 0.14 | 0.09 ± 0.13 |
| Bile flow ± s.d. (mg $min^{-1}$ $g^{-1}$) in 5-FU alone | 1.38 ± 0.14 | 1.10 ± 0.31 | 1.00 ± 0.18 | 0.68 ± 0.12 | 0.35 ± 0.10 | 0.17 ± 0.05 | 0.09 ± 0.05 | 0.08 ± 0.04 |
| Bile flow ± s.d. (mg $min^{-1}$ $g^{-1}$) in 5-FU + GW776 | 1.09 ± 0.08 | 0.88 ± 0.22 | 0.78 ± 0.09 | 0.56 ± 0.04 | 0.30 ± 0.04 | 0.13 ± 0.01 | 0.11 ± 0.01 | 0.08 ± 0.03 |

The purpose of the present study was thus to test the effects of GW776 on 5-FU metabolism, in particular with respect to formation of the toxic compounds FAC and FHPA, using fluorine-19 nuclear magnetic resonance ($^{19}$F-NMR) and the IPRL model.

### MATERIALS AND METHODS

### IPRL experiments

Livers from male Wistar rats (Iffa Credo, Lyon, France) were isolated and perfused with recirculation of the perfusion medium according to the design originally described by Brauer et al (1951) and modified by Sabouraud et al (1993). The recirculating perfusate (approximately 180 ml), oxygenated with oxygen–carbon dioxide (95%:5%), was a Krebs–Ringer bicarbonate buffer supplemented with glucose (1.3 mM) and bovine serum albumin (0.5%). The perfusate (pH 7.4) was recirculated at a mean flow rate of 10 ml $min^{-1}$ $g^{-1}$ of liver. The experiments were carried out with solutions of 5-FU prepared immediately before use at a dose of 15 mg $kg^{-1}$ body weight. For experiments with 5-FU alone, the drug was injected into the perfusate after 1 h of liver equilibration, and the experiments (*n* = 5) were continued for 3 h. For experiments with 5-FU + GW776, 5-FU was injected after 1 h of liver equilibration in the presence of GW776 at a dose of 0.5 mg $kg^{-1}$ body weight (Spector et al, 1993), and the experiments (*n* = 4) were continued for 3 h. A 0.5 M sodium bicarbonate solution was continuously infused into the reservoir to maintain the pH of the perfusate at 7.4. The temperature and pH of the perfusate, portal vein pressure and bile flow were continuously monitored. Bile was collected in preweighed vials at 30-min intervals after the beginning of the liver perfusion. Liver viability was assessed by measuring lactate dehydrogenase (LDH) activity in the perfusate every 30 min.

The pH, vascular resistance, oxygen consumption, LDH activity and bile flow were in the range of the literature values (Sugano et al, 1978; Van Dyke et al, 1983). The evolution of LDH activity and bile flow are shown in Table 1. There was no significant difference in values of LDH activity in control experiments and in experiments with 5-FU alone and with 5-FU + GW776. The bile flow was slightly weaker in experiments with 5-FU + GW776 than in control experiments and in experiments with 5-FU alone, but it was in the range of other experiments with 5-FU that are not reported here.

At the end of the experiments, an aliquot of the perfusate was immediately frozen and kept at – 80°C until $^{19}$F-NMR analysis. The remaining perfusate was freeze-dried and then resuspended in ~3 ml of water immediately before $^{19}$F-NMR analysis. Bile samples were gathered and stored at – 80°C until analysis. Liver was weighed, immersed in liquid nitrogen, powdered and sequentially extracted with cold and hot 1 M perchloric acid by using the method of Wain and Staatz (1973). The acid-soluble and acid-insoluble fractions thus obtained were lyophilized to dryness and stored at – 80°C until analysis. The lyophilized materials were then resuspended in approximately 2.5 ml of water containing 30 mM EDTA immediately before $^{19}$F-NMR analysis.

### Verification of the metabolic origin of FAC and FHPA

To check that the formation of FAC and FHPA was a metabolic process rather than a chemical transformation of 5-FU or FBAL taking place during the perfusion, several control experiments were carried out.

We first checked the purity of the solution of 5-FU injected and demonstrated that it was not chemically transformed in the perfusate bubbled with carbogen for 3 h at 37°C. Indeed, the $^{19}$F-NMR spectrum of the perfusion medium after concentration by freeze-drying displayed the single 5-FU peak. We also carried out four control experiments in which the perfusate containing 5-FU at a concentration of 45 mg $kg^{-1}$ body weight (calculated for a mean rat body weight of 400 g) was circulated for 3 h in the perfusion system without liver. The $^{19}$F-NMR spectra of these perfusates after concentration only exhibited a strong peak corresponding to

© Cancer Research Campaign 1997

5-FU, which made up 99.8 ± 0.07% of all the fluorinated compounds detected, the fluoride ion (F–) signal representing 0.05 ± 0.02% and two very weak signals at δ = – 111.3 and –111.6 p.p.m. (0.08 ± 0.05% and 0.03 ± 0.01% respectively) not corresponding to any of the signals observed in the concentrated perfusates from the IPRL experiments with 5-FU.

Two control experiments were carried out with FBAL. We first recorded the $^{19}$F-NMR spectrum of a solution of FBAL in the perfusion medium over a period of 3 h and only detected the signals of FBAL (98.9%) and N-carboxy-α-fluoro-β-alanine (CFBAL; 1.1%). We also carried out one control experiment in which the perfusate containing FBAL at a concentration of 16.6 mg kg$^{-1}$ body weight (dose equivalent to 15 mg 5-FU kg$^{-1}$ body weight and calculated for a mean rat body weight of 400 g) was circulated for 3 h in the perfusion system without liver. The $^{19}$F-NMR spectrum of this perfusate after concentration only displayed the signals of FBAL, which made up 18.3% of all the fluorinated compounds detected, CFBAL representing 49.6%, $F^-$ 1.4% and the adducts of FBAL with α-glucose 2.4% and β-glucose 28.3%.

### NMR spectroscopy

$^{19}$F-NMR spectra were recorded at 282.4 MHz with $^1$H-decoupling on a Bruker WB-AM 300 spectrometer in the following conditions: probe temperature, 25°C; sweep width, 41 667 Hz; 32 768 data points zero-filled to 65 536; pulse width, 7 μs (flip angle approximately 40° in non-concentrated perfusate and bile, approximately 30° in perchloric acid extracts and approximately 20° in concentrated perfusate); pulse interval, 1.4 s for quantification of concentrated perfusates, acid-soluble and acid-insoluble extracts or 3.4 s for quantification of non-concentrated perfusates and bile samples; number of scans, 10 000–50 000; line broadening caused by exponential multiplication, 6 Hz. The chemical shifts (δ) were reported relative to the resonance peak of trifluoroacetic acid (5% w/v aqueous solution) used as external chemical shift reference. The concentrations of the fluorinated compounds were measured by comparing the expanded areas of their NMR signals with that of the external standard for quantification placed in a coaxial capillary, namely a solution of sodium parafluorobenzoate (FBEN) in deuterium oxide doped at saturation with chromium(III) acetylacetonate (Cr(acac)$_3$) to shorten the longitudinal relaxation time ($T_1$) of FBEN. The apparent concentration of the FBEN peak was previously calibrated. Cr(acac)$_3$ (approximately 2.5 mg) was also added to non-concentrated perfusates and bile samples. The areas were determined after the different signals were cut out and weighed.

Fully relaxed spectra were obtained for all media analysed, even when spectra were recorded with a pulse interval as short as 1.4 s and without Cr(acac)$_3$. This was demonstrated for (1) 5-FU, FAC and FHPA in concentrated perfusates recorded with a pulse interval of 1.4 s or 3.4 s without Cr(acac)$_3$ or 10.4 s with Cr(acac)$_3$, (2) 5-FU and FBAL in acid-soluble extracts containing EDTA recorded with a pulse interval of 1.4 s without Cr(acac)$_3$ or 3.4 s with Cr(acac)$_3$. The differences between the values of concentrations thus determined were not more than 10%, which corresponds to the precision of the method (5–10% depending on the concentration; Malet-Martino and Martino, 1992). The high ionic strength of concentrated perfusates and perchloric acid extracts and the high viscosity of the former medium induced a decrease of the flip angle for a given value of the pulse width (see above) and probably of the $T_1$, leading to an accurate quantification even with a low pulse interval and without Cr(acac)$_3$.

As a step of lyophilization was necessary to measure FHPA and FAC concentrations, we checked the recovery of these two compounds from the lyophilization pellet. Known amounts of FHPA and FAC were added to 450 ml of blank perfusate at concentrations close to those found in the perfusates from rat liver experiments. Three 150-ml fractions were freeze-dried and taken up in water under our normal operating conditions. $^{19}$F-NMR spectroscopy showed that only 76 ± 2% and 63 ± 15% of FHPA and FAC, respectively, were recovered. The amounts of FHPA and FAC measured in our experiments were thus underestimated as material remained in the pellet, which was not completely redissolved.

We noticed that the amounts of FBAL (and derivatives) and $F^-$ measured in concentrated perfusates were lower than in non-concentrated perfusates. Of FBAL (and its derivatives), 71 ± 9% was recovered in concentrated perfusates of experiments with 5-FU alone, whereas only 46 ± 10% of $F^-$ was recovered in experiments with 5-FU alone and with 5-FU + GW776. This led us to quantify all the 5-FU metabolites in non-concentrated perfusates, except when they were not detected (FBAL and related compounds in 5-FU + GW776 experiments and FAC and FHPA in 5-FU experiments).

### Statistical analysis

All results were expressed as means ± s.d. When necessary, statistical significance was determined using Student's *t*-test. A *P*-value of $< 0.05$ was considered to be statistically significant.

### RESULTS

### Qualitative analysis

IPRL were treated with pure 5-FU at a 'therapeutic' dose of 15 mg kg$^{-1}$ body weight for 3 h with ($n = 4$) or without ($n = 5$) a 1-h prior treatment with GW776 at a dose of 0.5 mg kg$^{-1}$ body weight (Spector et al, 1993).

A characteristic $^{19}$F-NMR spectrum of a non-concentrated perfusate from an IPRL treated with 5-FU alone shows the large signals of 5-FU main catabolites, FBAL at δ = –112.4 p.p.m. and $F^-$ coming from the defluorination of FBAL (Martino et al, 1985; Porter et al, 1995) at δ = – 43.5 p.p.m. Low signals from α-fluoro-β-ureidopropionic acid (FUPA) at δ = – 110.7 p.p.m. and CFBAL derived from the interaction of bicarbonate ion with FBAL (Martino et al, 1987) at δ = – 110.9 p.p.m. could also be detected (Figure 1A). A large signal of 5-FU at δ = – 93.3 p.p.m. and a weak resonance for $F^-$ were observed in the $^{19}$F-NMR spectrum of a non-concentrated perfusate from an IPRL treated with 5-FU + GW776 (Figure 1B). $FUH_2$ was not observed in any of the experiments.

A characteristic $^{19}$F-NMR spectrum of a concentrated perfusate from an IPRL treated with 5-FU alone (Figure 2A) shows the signals of FBAL at δ = – 112.6 p.p.m., CFBAL at δ = – 111.4 p.p.m. and $F^-$ at δ = – 49.0 p.p.m. The differences in the values of chemical shifts in non-concentrated and concentrated perfusates are mainly due to the much higher ionic strength in the concentrated perfusates and to differences in pH (7.6 vs 8.3 respectively). More CFBAL was present in the concentrated perfusates (compare Figures 1A and 2A) as the proportion of CFBAL with respect to

© Cancer Research Campaign 1997

**Figure 1** $^{19}$F-NMR spectra of a non-concentrated perfusate from an isolated perfused rat liver treated (**A**) with 5-FU-alone (15 mg kg$^{-1}$ body weight) or (**B**) with 5-FU + GW776 (15 mg 5-FU kg$^{-1}$ body weight and 0.5 mg GW776 kg$^{-1}$ body weight injected 1 h before 5-FU). (**A**) pH 7.4. (**B**) pH 7.5

FBAL increases with pH up to about pH 9 (Martino et al, 1987). The strong resonance at δ = – 111.0 p.p.m. and the weak signal at δ = – 110.4 p.p.m. are artifacts of freeze-drying. These two signals only appear in the concentrated perfusates and correspond to adducts of metabolic FBAL in the *R* configuration (Duschinsky et al, 1973; Gani et al, 1985) with β-glucose (FBAL[R]-glucβ, δ = – 111.0 p.p.m.) and α-glucose (FBAL[R]-glucα, δ = – 110.4 p.p.m.). The two signals at δ = – 113.6 p.p.m. and – 141.3 p.p.m. were assigned to FHPA and FAC, respectively, and were positively identified by spiking a perfusate with authentic standards. The control experiments described in Materials and methods showed unambiguously that FAC and FHPA did not arise from a chemical transformation of 5-FU or FBAL taking place during the perfusion experiment or the freeze-drying step but were formed via a metabolic process. In a $^{19}$F-NMR spectrum of a concentrated perfusate from an IPRL treated with 5-FU + GW776 (Figure 2B), a large signal of 5-FU at δ = – 93.2 p.p.m. and low signals from FBAL[R]-glucβ, CFBAL, FBAL, $F^-$ and 5-fluorouridine (FUR) at δ = – 88.0 p.p.m. were observed.

In bile samples from experiments with 5-FU alone (Figure 3A), the resonances of FBAL (δ = – 112.4 p.p.m.) and $F^-$ (δ = – 43.5 p.p.m.) were observed together with those of FBAL

**Figure 2** $^{19}$F-NMR spectra of a concentrated perfusate from an isolated perfused rat liver treated (**A**) with 5-FU alone (15 mg $kg^{-1}$ body weight) or (**B**) with 5-FU + GW776 (15 mg 5-FU $kg^{-1}$ body weight and 0.5 mg GW776 $kg^{-1}$ body weight injected 1 h before 5-FU). (**A**) pH 8.3. (**B**) pH 8.4

conjugates with bile acids, most probably the conjugates with cholic, deoxycholic and muricholic acids ($\delta$ = – 110.3, – 110.7 p.p.m. and – 110.9 p.p.m., the last signal being low and observed in only two out of five experiments) (Malet-Martino et al, 1988; Sweeny et al, 1988). A characteristic $^{19}$F-NMR spectrum of a bile sample from an experiment with 5-FU + GW776 only exhibited the signal of $F^-$ (Figure 3B).

In the acid-soluble extract of liver treated with 5-FU alone, the major signal corresponded to FBAL ($\delta$ = – 112.3 p.p.m.). The other resonances were those of fluoronucleotides (FNUCts; $\delta$ = – 89.2 p.p.m.), 5-fluorouridine-5′-diphosphate sugars (FUDP sugars; $\delta$ = – 89.1 p.p.m.) and unknown compounds ($\delta$ = – 93.8, – 110.1, – 110.4 and – 110.8 p.p.m.), which probably arose from chemical degradation of FBAL occurring during the step in very acidic medium necessary to extract the liver (Figure 4A). The main signal found in the acid-soluble extract of liver treated with 5-FU + GW776 was that of 5-FU ($\delta$ = – 93.4 p.p.m.). FNUCts and FUDP sugars led to four well-resolved resonances at $\delta$ = – 89.04, – 89.07, – 89.16 and – 89.27 p.p.m. Other signals were those of FUR ($\delta$ = – 90.0 p.p.m.), FBAL and the unknown compound resonating at $\delta$ = – 93.8 p.p.m. (Figure 4B).

The signal of FBAL ($\delta$ = – 112.3 p.p.m.) was the only one detected in the acid-insoluble extract of liver treated with 5-FU alone (Figure 5A). It probably came from the incomplete extraction of the acid-soluble fraction. On the other hand, the $^{19}$F-NMR spectrum of an acid-insoluble extract of a liver treated with

 © Cancer Research Campaign 1997

**Figure 3** $^{19}$F-NMR spectra of a bile sample from an isolated perfused rat liver treated (**A**) with 5-FU alone (15 mg $kg^{-1}$ body weight) or (**B**) with 5-FU + GW776 (15 mg 5-FU $kg^{-1}$ body weight and 0.5 mg GW776 $kg^{-1}$ body weight injected 1 h before 5-FU). (**A** and **B**) pH 8.5.

5-FU + GW776 showed the signals of 5-fluorouridine-2′-monophosphate (2′-FUMP; δ = – 89.5 p.p.m.) and 5-fluorouridine-3′-monophosphate (3′-FUMP; δ = – 89.8 p.p.m.), which arose from the acidic hydrolysis at 70°C of 5-FU incorporated into RNA (Parisot et al, 1991). A low signal of 5-fluorouridine-5′-monophosphate (5′-FUMP; δ = – 89.2 p.p.m.) and the resonance of 5-FU (δ = – 93.4 p.p.m.) were also observed (Figure 5B). These compounds probably came from the incomplete extraction of the acid-soluble fraction.

### Quantitative analysis

### *Global recovery of 5-FU and its metabolites*

The data are presented in Table 2. The global recovery is identical (approximately 67%) in the two sets of experiments. We checked the error of the $^{19}$F-NMR assay by adding a known amount of 5-FU to 150 ml of blank perfusate (final concentration $9.5 \times 10^{-4}$ M) and by dosing three separate aliquots. $^{19}$F-NMR spectroscopy showed that 95.0 ± 0.7% of 5-FU was recovered. The $^{19}$F-NMR assay is therefore accurate and the error made on it cannot explain the missing amount of drug and/or metabolites in liver experiments. We noticed that there was an evaporation of perfusate during the 4 h of perfusion. To quantify this loss, we carried out 12 control experiments as follows. The perfusate containing 5-FU at a concentration of 15 or 45 mg $kg^{-1}$ body weight (calculated for a mean rat body weight of 400 g), or FBAL or 5′-deoxy-5-fluorouridine at a concentration equivalent to 15 or 45 mg 5-FU $kg^{-1}$ body weight, respectively, was circulated for 3 h in the perfusion system without liver. The mean volume of perfusate lost during these experiments was 38.9 ± 5.6 ml. This loss

© Cancer Research Campaign 1997

**Figure 4** $^{19}$F-NMR spectra of an acid-soluble extract from an isolated perfused rat liver treated (**A**) with 5-FU alone (15 mg $kg^{-1}$ body weight) or (**B**) with 5-FU + GW776 (15 mg 5-FU $kg^{-1}$ body weight and 0.5 mg GW776 $kg^{-1}$ body weight injected 1 h before 5-FU). (**A** and **B**) pH 5.4

was due to the temperature in the perfusion device, the high speed of perfusate recirculation and the high flow rate of carbogen bubbling, both necessary to insure a correct oxygenation of the liver. The mean recovery of fluorinated compound was 85.6 ± 3.1%, clearly demonstrating that approximately 15% of the injected dose of 5-FU, FBAL or 5′-deoxy-5-fluorouridine was evaporated with the perfusate during the liver perfusion. The remaining missing drug and/or metabolites (approximately 19%) probably stayed in the liver that was not completely extracted with the extraction methodology used in our study.

All 5-FU was metabolized in experiments with 5-FU alone, whereas 64% of the injected 5-FU (i.e. approximately 94% of the fluorinated compounds measured) was recovered unchanged in experiments with 5-FU + GW776. The amount of 5-FU catabolites decreased by a factor of 27 in the presence of GW776, whereas 5-FU anabolites increased by a factor of 7.

### *Proportions of 5-FU and its metabolites*

The data are presented in Table 3. In the experiments with 5-FU alone, FBAL was the main catabolite as it represented 44% of the 5-FU-injected dose whereas $F^-$, present in trace amounts, was the main catabolite (2% of injected dose) in the experiments with 5-FU + GW776.

The main characteristics of the experiments with 5-FU + GW776 are (1) the decrease in FBAL formation by a factor of ~110, (2) the absence of FAC and FHPA formation [although only

© Cancer Research Campaign 1997

**Figure 5** $^{19}$F-NMR spectra of an acid-insoluble extract from an isolated perfused rat liver treated (**A**) with 5-FU alone (15 mg kg$^{-1}$ body weight) or (**B**) with 5-FU + GW776 (15 mg 5-FU kg$^{-1}$ body weight and 0.5 mg GW776 kg$^{-1}$ body weight injected 1 h before 5-FU). (**A** and **B**) pH 5.4

small amounts of FHPA and FAC were found in the perfusates of experiments with 5-FU alone (FHPA represented 0.4% and FAC 0.1% of the injected 5-FU)] and (3) the increase by a factor of approximately 5 of FNUCts and FUDP sugars and the low but measurable incorporation of 5-FU into RNA.

### *Repartition of 5-FU and metabolites in the different media analysed (perfusate, bile and liver)*

The data presented in Table 4 indicate that, for all experiments, most of the fluorinated compounds were found in the perfusate. Almost identical amounts of fluorinated compounds were measured in bile samples and acid-insoluble extracts. On the other hand, the proportion of fluorinated compounds is much higher in acid-soluble extracts from experiments with 5-FU alone (13%) than from experiments with 5-FU + GW776 (≈3%).

FBAL and $F^-$ were the main compounds in perfusates from experiments with 5-FU alone (making up, respectively, approximately 58% and approximately 40% of the fluorinated compounds measured in perfusates), whereas unmetabolized 5-FU represented approximately 97% in perfusates from experiments with 5-FU + GW776. Only 5-FU catabolites were found in bile samples, essentially FBAL and FBAL conjugates in 5-FU-alone experiments and only $F^-$ in 5-FU + GW776 experiments. Although the catabolic pathway was reduced by a factor of 27 when 5-FU was injected in association with GW776, the trace amount of $F^-$ was about five-fold higher in bile samples from experiments with 5-FU + GW776.

© Cancer Research Campaign 1997

**Table 2** Global recovery of 5-FU and metabolites in experiments with 5-FU alone and with 5-FU + GW776

| Compounds | Per cent of injected dose ± s.d. | |
|---|---|---|
| | Experiments with 5-FU alone | Experiments with 5-FU + GW776 |
| Unmetabolized 5-FU | 0 | 64 ± 3 |
| Catabolites | 66 ± 5 | 2.4 ± 0.8 |
| Anabolites | 0.16 ± 0.04[a] | 1.1 ± 0.3[a] |
| Total | 66 ± 5[b] | 68 ± 4[b] |

[a] Significant at $P < 0.0005$. [b]Not significant ($P > 0.1$).

**Table 3** Proportions of 5-FU and metabolites in experiments with 5-FU alone and with 5-FU + GW776

| Compounds | Percent of injected dose ± s.d. | |
|---|---|---|
| | Experiments with 5-FU alone | Experiments with 5-FU + GW776 |
| Unmetabolized 5-FU | 0 | 64 ± 3 |
| Catabolites | | |
| FUPA | 0.6 ± 0.7 | 0 |
| FBAL | 44 ± 11[a] | 0.4 ± 0.2[b] |
| $F^-$ | 22 ± 7 | 2 ± 1 |
| FAC[c] | 0.11 ± 0.04 | 0 |
| FHPA[c,d] | 0.42 ± 0.06 | 0 |
| Anabolites | | |
| 5′ FNUCt and FUDP sugars | 0.16 ± 0.04[e] | 0.8 ± 0.4[e] |
| FU-RNA (2′ and 3′ FUMP) | 0 | 0.09 ± 0.01 |
| FUR | 0[f] | 0.3 ± 0.1 |

[a]FBAL includes FBAL and CFBAL in non-concentrated perfusate, FBAL and FBAL conjugates with bile acids in bile, FBAL and unknown compounds (δ = – 93.8, – 110.1, – 110.4 and – 110.8 p.p.m.) in acid-soluble extract and FBAL in acid-insoluble extract. [b]FBAL includes FBAL, CFBAL, FBAL[R]-glucβ in concentrated perfusate, and FBAL and unknown compound (δ = – 93.8 p.p.m.) in acid-soluble extract. [c]FAC and FHPA could only be assayed in concentrated perfusate. [d]The signal of FHPA in non-concentrated perfusate is in the base of the large signal of FBAL; it is thus already included when FBAL signal is assayed. [e]Significant at $P < 0.01$. [f]Only observed in one experiment out of five, representing 0.02% of injected dose.

The amount of 5-FU measured in acid-soluble extracts from experiments with 5-FU + GW776 was low compared with that of FBAL in acid-soluble extracts from experiments with 5-FU alone, demonstrating that 5-FU, contrary to FBAL, is not stored into hepatocytes. No anabolites were detected in acid-insoluble extracts from 5-FU-alone experiments, whereas the experiments with 5-FU + GW776 demonstrated a low incorporation of 5-FU into RNA of hepatocytes.

### DISCUSSION

GW776 modulates both the catabolic and anabolic pathways of 5-FU, the most striking effect being on the degradative pathway. The amount of 5-FU catabolites decreased by a factor of 27 in the presence of GW776 (Table 2). Rat livers were thus > 96% inhibited in their ability to catalyse 5-FU degradation. This result is in complete agreement with the study of Baccanari et al (1993), which reported the same extent of inhibition when DPD activity was measured in liver extracts prepared 1 h or 6 h after rats were treated with a single dose of GW776 (2 mg $kg^{-1}$ p.o.).

GW776 led to a decrease in FBAL formation by a factor of ~110, whereas $F^-$ formation decreased by a factor of only ~10 (Table 3). L-alanine-glyoxylate aminotransferase II (AlaAT-II) purified from rat liver catalysed the elimination of $F^-$ from FBAL. The enzyme was not inactivated significantly during dehalogenation of FBAL, and 5-FU was not a substrate (Porter et al, 1995). To explain our data, one might therefore evoke a slight defluorination of the large amounts of unmetabolized 5-FU remaining during the experiments with 5-FU + GW776 catalysed by another enzyme.

FAC is a highly cardiotoxic and neurotoxic poison (Pattison and Peters, 1966) known to accumulate in the organism (Meldrum and Bignell 1957). We checked the cardiotoxicity of FHPA on the isolated perfused rabbit heart model at two doses, 0.01 and 1.5 mg $kg^{-1}$. FHPA did not generate cardiotoxic symptoms at the lowest dose but was highly cardiotoxic on this model at the highest dose (unpublished data). Moreover, FBAL, the precursor of FHPA and FAC (Arellano et al, 1994), accumulated in rats and was retained up to 8 days in various tissues, mainly liver, heart and brain (Zhang et al, 1992, 1993). By greatly lowering the metabolization of 5-FU into FBAL, GW776 circumvents the formation of toxic FHPA and FAC (Table 3). The present results therefore support the earlier report of Davis et al (1994). These authors demonstrated that GW776 protected dogs from the neurotoxicity induced by a 26-h continuous infusion of 5-FU at three doses (1.6, 4 or 16 mg $kg^{-1}$ 24 $h^{-1}$) and suggested with others (Koenig and Patel, 1970; Okeda et al, 1984, 1990) that 5-FU catabolites are responsible for this dose-limiting toxicity in dogs and cats, which are particularly sensitive.

© Cancer Research Campaign 1997

**Table 4** Repartition of 5-FU and metabolites in perfusate, bile and liver (acid-soluble and acid-insoluble extracts) from experiments with 5-FU alone and with 5-FU + GW776

| **Medium and compound** | **Per cent of injected dose ± s.d.** | |
|---|---|---|
| | **Experiments with 5-FU alone** | **Experiments with 5-FU + GW776** |
| Perfusate | | |
| 5-FU | 0 | 62 ± 3 |
| FUR | 0 | 0.2 ± 0.1[a] |
| FUPA | 0.6 ± 0.7 | 0 |
| FBAL | 30 ± 9[b] | 0.12 ± 0.07[a,c] |
| $F^-$ | 21 ± 7 | 1.6 ± 0.8 |
| FAC[a] | 0.11 ± 0.04 | 0 |
| FHPA[a] | 0.42 ± 0.06 | 0 |
| Total | 52 ± 5[d,e] | 64 ± 4[e] |
| Bile | | |
| FBAL | 0.13 ± 0.04 | 0 |
| FBAL conjugates | 0.3 ± 0.2 | 0 |
| $F^-$ | 0.07 ± 0.02[f] | 0.4 ± 0.3[f] |
| Total | 0.5 ± 0.2[g] | 0.4 ± 0.3[g] |
| Acid-soluble extract | | |
| 5-FU | 0 | 2.3 ± 0.6 |
| FUR | 0[h] | 0.03 ± 0.006 |
| FNUCts | 0.16 ± 0.04[e] | 0.75 ± 0.4[e] |
| FBAL | 12.5 ± 5 | 0.2 ± 0.1 |
| Unknown compound(s) | 0.45 ± 0.06[i] | 0.09 ± 0.04[j] |
| Total | 13 ± 4[e] | 3.4 ± 1.1[e] |
| Acid-insoluble extract | | |
| 5-FU | 0 | 0.09 ± 0.10 |
| 5′, 2′ and 3′ FUMP | 0 | 0.10 ± 0.03 |
| FBAL | 0.17 ± 0.08 | 0 |
| Total | 0.17 ± 0.08[g] | 0.19 ± 0.12[g] |

[a]FUR, FBAL, FAC and FHPA could only be assayed in concentrated perfusate. [b]FBAL includes FBAL and CFBAL. [c]FBAL includes FBAL, CFBAL and FBAL[R]-glucβ. [d]The signal of FHPA in non-concentrated perfusate is in the base of the large signal of FBAL; FHPA is thus assayed at the same time as FBAL. This explains why the total does not include the value found for FHPA. [e]Significant at $P < 0.01$. [f]Significant at $P < 0.025$. [g]Not significant ($P > 0.1$). [h]Only observed in one experiment out of five, representing 0.02% of injected dose. [i]The unknown compounds resonate at δ = – 93.8, – 110.1, – 110.4 and – 110.8 p.p.m. [j]Only the unknown compound resonating at δ = – 93.8 p.p.m. could be detected.

Also interesting is the effect of GW776 on the anabolic pathway of 5-FU that confers to the drug its cytotoxicity. GW776 increases the therapeutic index of 5-FU in mouse (Baccanari et al, 1993) and rat (Cao et al, 1994) tumour models. As GW776 prevents the catabolism of 5-FU and thus improves systemic exposure to 5-FU, one might expect an increase in the amounts of 5-FU anabolites to explain the better efficacy. The liver is not the best model to study 5-FU anabolism. Moreover, the comparison between our experiments with 5-FU alone and with 5-FU + GW776 would have been more convincing with a lower dose of 5-FU when injected in combination with GW776. We used the present dose to be able to detect low amounts of catabolites. Nevertheless, our results demonstrate that 5-FU anabolites increased by a factor of ~7 in the presence of GW776 (Table 2). The level of free FNUCts and FUDP sugars was increased up to ~ fivefold (Table 3). No 5-FU incorporated into RNA was observed in the experiments with 5-FU alone whereas the incorporation of 5-FU into RNA, detected as 2′- and 3′FUMP, was low (0.1% of the injected dose) but detectable in the experiments with 5-FU + GW776 (Table 3). Davis et al (1995) reported that, compared with mice treated with 5-FU alone, GW776 enhanced the incorporation of 5-FU into RNA in MOPC-315 s.c. tumours of mice.

In conclusion, our study clearly shows that GW776 prevents formation of FBAL and its subsequent metabolism into the toxic FHPA and FAC. GW776 may therefore be useful not only for improving the efficacy of 5-FU (Spector et al, 1994) but also for attenuating cardiotoxic and/or neurotoxic side-effects of this antitumour agent that may be due to FBAL metabolites. Although these side-effects are not very common, they are severe and may be dose limiting. The combination of GW776 and 5-FU has great clinical potential. GW776 increases the half-life of 5-FU from approximately 14 min to approximately 5 h in humans (Khor et al, 1996) and thereby enables oral dosing to replace the 5-day bolus and the protracted continuous infusion schedules (Baccanari et al, 1993). Moreover, the dry powder of orally formulated 5-FU presents the advantage of being devoid of cardiotoxic breakdown products found in i.v. solutions (Lemaire et al, 1992). This combination is currently in international phase II clinical trials for breast, pancreatic, colorectal and hepatocellular cancer.

### ABBREVIATIONS

5-FU, 5-fluorouracil; FBAL, α-fluoro-β-alanine; FAC, fluoroacetate; FMASAld, fluoromalonic acid semi-aldehyde; Facet, fluoroacetaldehyde; FHPA, α-fluoro-β-hydroxypropionic acid; IPRL, isolated perfused rat liver; GW776, 5-ethynyluracil; DPD, dihydropyrimidine dehydrogenase; $FUH_2$, 5,6-dihydro-5-fluorouracil; $^{19}$F-NMR, fluorine-19 nuclear magnetic resonance; LDH,

© Cancer Research Campaign 1997 

lactate dehydrogenase; EDTA, ethylene diamine tetraacetic acid; $F^-$, fluoride ion; CFBAL, N-carboxy-α-fluoro-β-alanine; FBEN, sodium parafluorobenzoate; $Cr(acac)_3$, chromium(III) acetylacetonate; FUPA, α-fluoro-β-ureidopropionic acid; FBAL [R]-glucβ, FBAL [S]-glucβ, FBAL [R]-glucα, FBAL [S]-glucα, adducts of FBAL with β-glucose and α-glucose; FUR, 5-fluorouridine; FNUCts, fluoronucleotides; FUDP sugars, 5-fluorouridine-5′-diphosphate sugars; 2′-FUMP, 5-fluorouridine-2′-monophosphate; 3′FUMP, 5-fluorouridine-3′-monophosphate; 5′FUMP, 5-fluorouridine-5′-monophosphate; AlaAT-II, L-alanine-glyoxylate aminotransferase

### ACKNOWLEDGEMENTS

This work was supported by grant 6635 from the Association pour la Recherche sur le Cancer (to RM) and by grant from Ligue Nationale Française contre le Cancer (Section des Hautes-Pyrénées) to MA.

### REFERENCES

Anand AJ (1994) Fluorouracil cardiotoxicity. *Ann Pharmacother* **28**: 374–378

Arellano M, Malet-Martino M and Martino R (1994) First demonstration that the anticancer drug, 5-fluorouracil, is metabolized in the isolated perfused rat liver into highly cardiotoxic fluoroacetate. *Proc Soc Magnetic Resonance* 1320.

Baccanari DP, Davis ST, Knick VC and Spector T (1993) 5-ethynyluracil (776C85): a potent modulator of the pharmacokinetics and antitumor efficacy of 5-fluorouracil. *Proc Natl Acad Sci USA* **90**: 11064–11068

Bernadou J, Armand JP, Lopez A, Malet-Martino MC and Martino R (1985) Complete urinary excretion profile of 5-fluorouracil during a six-day chemotherapeutic schedule, as resolved by $^{19}$F nuclear magnetic resonance. *Clin Chem* **31**: 846–848

Brauer RW, Pessotti RL and Pizzolato P (1951) Isolated rat liver preparation. Bile production and other basic properties. *Proc Soc Exp Biol Med* **78**: 174–181

Cao S, Rustum YM and Spector T (1994) 5-ethynyluracil (776C85): modulation of 5-fluorouracil efficacy and therapeutic index in rats bearing advanced colorectal carcinoma. *Cancer Res* **54**: 1507–1510

Davis ST, Joyner SS, Baccanari DP and Spector T (1994) 5-ethynyluracil (776C85): protection from 5-fluorouracil-induced neurotoxicity in dogs. *Biochem Pharmacol* **48**: 233–236

Davis ST, Baccanari DP and Spector T (1995) 5-ethynyluracil (5EU, 776C85): effects on thymidylate synthase (TS) inhibition and incorporation of 5-FU into RNA in mice bearing s.c. MOPC-315 tumors. *Proc Am Assoc Cancer Res* **36**: 292

Duschinsky R, Walker H, Wojnarowski W, Noack K and Bächtold HP (1973) (+)-α-fluoro-β-alanine (FBAL), the main metabolite of 5-fluorouracil (FU) and its enantiomer. *Proc Am Assoc Cancer Res* **14**: 109

Gani D, Hitchcock PB and Young DW (1985) Stereochemistry of catabolism of the DNA base thymine and of the anti-cancer drug 5-fluorouracil. *J Chem Soc Perkin Trans l*, 1363–1372

Heggie GD, Sommadossi JP, Cross DS, Huster WJ and Diasio RB (1987) Clinical pharmacokinetics of 5-fluorouracil and its metabolites in plasma, urine, and bile. *Cancer Res* **47**: 2203–2206

Hull WE, Port RE, Herrmann R, Britsch B and Kunz W (1988) Metabolites of 5-fluorouracil in plasma and urine, as monitored by $^{19}$F nuclear magnetic resonance spectroscopy, for patients receiving chemotherapy with or without methotrexate pretreatment. *Cancer Res* **48**: 1680–1688

Khor SP, Lucas S, Hsieh AY, Schilsky R, Burris H, Von Hoff DD and Spector T (1996) 776C85: effect on renal elimination of 5-fluorouracil and uracil in cancer patients. *Proc Am Assoc Cancer Res* **37**: 371

Koenig H and Patel A (1970) Biochemical basis for fluorouracil neurotoxicity. *Arch Neurol* **23**: 155–160

Lemaire L, Malet-Martino MC, De Forni M, Martino R and Lasserre B (1992) Cardiotoxicity of commercial 5-fluorouracil vials stems from the alkaline hydrolysis of this drug. *Br J Cancer* **66**: 119–127

Lemaire L, Malet-Martino MC, Martino R, De Forni M and Lasserre B (1994) The Tris formulation of 5-fluorouracil is more cardiotoxic than the sodium salt formulations. *Oncol Rep* **1**: 173–174

Lemaire L, Arellano M, Malet-Martino M and Martino R (1996) A novel metabolite of 5-fluorouracil in humans: 2-fluoro-3-hydroxypropionic acid. *Proc Am Assoc Cancer Res* **37**: 1225

Malet-Martino MC and Martino R (1992) Magnetic resonance spectroscopy: a powerful tool for drug metabolism studies. *Biochimie* **74**: 785–800

Malet-Martino MC, Bernadou J, Martino R and Armand JP (1988) $^{19}$F NMR spectrometry evidence for bile acid conjugates of α-fluoro-β-alanine as the main biliary metabolites of antineoplastic fluoropyrimidines in humans. *Drug Metab Dispos* **16**: 78–84

Martino R, Lopez A, Malet-Martino MC, Bernadou J and Armand JP (1985) Release of fluoride ion from 5′-deoxy-5-fluorouridine, an antineoplastic fluoropyrimidine, in humans. *Drug Metab Dispos* **13**: 116–118

Martino R, Malet-Martino MC, Vialaneix C, Lopez A and Bon M (1987) $^{19}$F NMR analysis of the carbamate reaction of α-fluoro-β-alanine, the major catabolite of fluoropyrimidines. Application to FBAL carbamate determination in body fluids of patients treated with 5′-deoxy-5-fluorouridine. *Drug Metab Dispos* **15**: 897–904

Meldrum GK and Bignell JT (1957) The use of sodium fluoroacetate (compound 1080) for the control of the rabbit in Tasmania. *The Aust Vet J* **33**: 186–196

Mukherjee KL and Heidelberger C (1960) Studies on fluorinated pyrimidines. IX. The degradation of 5-fluorouracil-6-$C^{14}$. *J Biol Chem* **235**: 433–437

Okeda R, Karakama T, Kimura S, Toizumi S, Mitsushima T and Yokoyama Y (1984) Neuropathologic study on chronic neurotoxicity of 5-fluorouracil and its masked compounds in dogs. *Acta Neuropathol* **63**: 334–343

Okeda R, Shibutani M, Matsuo T, Kuroiwa T, Shimokawa R and Tajima T (1990) Experimental neurotoxicity of 5-fluorouracil and its derivatives is due to poisoning by the monofluorinated organic metabolites, monofluoroacetic acid and α-fluoro-β-alanine. *Acta Neuropathol* **81**: 66–73

Parisot D, Malet-Martino MC, Martino R and Crasnier P (1991) $^{19}$F nuclear magnetic resonance analysis of 5-fluorouracil metabolism in four differently pigmented strains of *Nectria haematococca*. *Appl Environ Microbiol* **57**: 3605–3612

Pattison FLM and Peters RA (1966) Monofluoro aliphatic compounds. In *Handbook of Experimental Pharmacology*, Smith FA (ed.), pp. 387–458. Springer: New York

Porter DJT, Chestnut WG, Merrill BM and Spector T (1992) Mechanism-based inactivation of dihydropyrimidine dehydrogenase by 5-ethynyluracil. *J Biol Chem* **267**: 5236–5242

Porter DJT, Harrington JA, Almond MR, Chestnut WG, Tanoury G and Spector T (1995) Enzymatic elimination of fluoride from α-fluoro-β-alanine. *Biochem Pharmacol* **50**: 1475–1484

Sabouraud A, Redureau M, Gires P, Martinet M and Scherrmann JM (1993) Effect of colchicine-specific Fab fragments on the hepatic clearance of colchicine. *Drug Metab Dispos* **21**: 997–1002

Spector T, Harrington JA and Porter DJT (1993) 5-ethynyluracil (776C85): inactivation of dihydropyrimidine dehydrogenase *in vivo*. *Biochem Pharmacol* **46**: 2243–2248

Spector T, Porter DJT, Nelson DJ, Baccanari DP, Davis ST, Almond MR, Khor SP, Amyx H, Cao S and Rustum YM (1994) 5-ethynyluracil (776C85), a modulator of the therapeutic activity of 5-fluorouracil. *Drugs Future* **19**: 565–571

Sugano T, Suda K, Shimada M and Oshino N (1978) Biochemical and ultrastructural evaluation of isolated rat liver systems perfused with a hemoglobin-free medium. *J Biochem* **83**: 995–1007

Sweeny DJ, Barnes S and Diasio RB (1988) Formation of conjugates of 2-fluoro-beta-alanine and bile acids during the metabolism of 5-fluorouracil and 5-fluoro-2-deoxyuridine in the isolated perfused rat liver. *Cancer Res* **48**: 2010–2014

Van Dyke RW, Gollan JL and Scharschmidt BF (1983) Oxygen consumption by rat liver: effects of taurocholate and sulfobromophthalein transport, glucagon, and cation substitution. *Am J Physiol* **244**: G523–G531

Wain WH and Staatz WD (1973) Rates of synthesis of ribosomal protein and total ribonucleic acid through the cell cycle of the fission yeast *Schizosaccharomyces pombe*. *Exp Cell Res* **81**: 269–278

Yeh KH and Cheng AL (1994) Acute confusion induced by a high-dose infusion of 5-fluorouracil and folinic acid. *J Formos Med Assoc* **93**: 721–723

Zhang R, Soong SJ, Liu T, Barnes E and Diasio RB (1992) Pharmacokinetics and tissue distribution of 2-fluoro-β-alanine in rats. Potential relevance to toxicity pattern of 5-fluorouracil. *Drug Metab Dispos* **20**: 113–119

Zhang R, Liu T, Soong SJ and Diasio RB (1993) A mathematical model of the kinetics and tissue distribution of 2-fluoro-β-alanine, the major catabolite of 5-fluorouracil. *Biochem Pharmacol* **45**: 2063–2069

© Cancer Research Campaign 1997